# Detection of Partial Discharge Sources Using UHF Sensors and Blind Signal Separation

**DOI:** 10.3390/s17112625

**Published:** 2017-11-15

**Authors:** Carlos Boya, Guillermo Robles, Emilio Parrado-Hernández, Marta Ruiz-Llata

**Affiliations:** 1 Department of Electronic Technology, Universidad Carlos III de Madrid, Avda, Universidad, 30, 28911 Leganés, Madrid, Spain; carlosallanb@gmail.com; 2 Department of Electrical Engineering, Universidad Carlos III de Madrid, Avda, Universidad, 30, 28911 Leganés, Madrid, Spain; grobles@ing.uc3m.es; 3 Department of Signal Processing and Communications, Universidad Carlos III de Madrid, Avda, Universidad, 30, 28911 Leganés, Madrid, Spain; eparrado@ing.uc3m.es

**Keywords:** blind source separation, electric insulation, partial discharges, UHF detection

## Abstract

The measurement of the emitted electromagnetic energy in the UHF region of the spectrum allows the detection of partial discharges and, thus, the on-line monitoring of the condition of the insulation of electrical equipment. Unfortunately, determining the affected asset is difficult when there are several simultaneous insulation defects. This paper proposes the use of an independent component analysis (ICA) algorithm to separate the signals coming from different partial discharge (PD) sources. The performance of the algorithm has been tested using UHF signals generated by test objects. The results are validated by two automatic classification techniques: support vector machines and similarity with class mean. Both methods corroborate the suitability of the algorithm to separate the signals emitted by each PD source even when they are generated by the same type of insulation defect.

## 1. Introduction

One of the causes of power failures and blackouts is the breakdown of the insulation systems of electric assets, produced, in some cases, by their deterioration. This aging can be premature and originates due to small and persistent discharges, called partial discharges (PDs). Thus, their measurement is crucial in the monitoring and maintenance of electric equipment.

One of the most common methods of PD detection is the measurement of the electromagnetic radiation emitted by the discharge. Therefore, monitoring is done with antennas with the capability of performing online supervision of wide areas in substations and aerial power lines [1,2,3]. However, automatic monitoring using these antenna based systems is hampered by the presence of series of pulses coming from different sources of electromagnetic emissions derived from interferences or several PD sources. This problem has to be faced in the post-processing of the received data to separate the pulses coming from each of the involved processes [4,5]. In this paper, we propose the application of independent component analysis (ICA) to distinguish the signals originating in each defect. The ICA approach has been widely used for blind source separation (BSS) in applications where a mixture of statistically independent sources is decomposed into individual source components [6]. ICA has been extensively applied in wireless communications [7], audio and speech signal processing  [8], and many other applications based on sensor array [9]. In the field of partial discharge detection, ICA has been proposed to separate mixed PD signals using the acoustic detection method [10], to separate UHF signals mixed synthetically in gas-isolated switchgear (GIS) [11] and to determine the time of arrival for PD location using a UHF detection method [12].

In this paper, we test the proposed ICA algorithm with real measurements and with the PDs of the same type that are generated in different places and detected with the same antennas. The algorithm is able to separate the individual signals from the two PD processes. To corroborate the results, a validation technique based on a supervised classification effectively confirms that the signals recovered by ICA accurately match those originating from the sources. This validation was carried out using two automatic classification techniques: support vector machines (SVMs) [13,14] and a naive approach consisting of classifying each datum with the class of the closest mean. These experiments show that the reconstructions achieved by ICA are very close to the original signals, therefore validating the proposed approach.

The rest of the paper is organized as follows: in Section 2 we present a brief introduction to partial discharges and the experimental setup used to generate the signals. Section 3 describes the theory underlying the proposed ICA algorithm. Section 4 contains the review of the automatic classification techniques used in the validation of ICA. In Section 5 we show the results with their validation, and the paper ends with conclusions in Section 6.

## 2. Partial Discharge Detection

Electrical assets can have parts of their insulation system susceptible to premature aging. Gas fills voids inside dielectrics, vacuoles in the paper-oil insulation in power transformers, or the cross-linked polyethylene (XLPE) in HV cables. Contaminated surfaces or interfaces among different materials such as capacitor bushings or sharp metallic geometries surrounded by air or gases are prone to partial discharges. In these sites, the air is easily and quickly ionized because the intensity of the electric field is higher than in the surrounding insulation system. This electric stress produces a series of small electric discharges that causes harmful aging processes that gradually debilitate the dielectric and bring about the ultimate failure even at operational voltages. Identifying this critical activity is paramount for preventing breakdowns and performing reliable maintenance of the asset, for instance, through the electromagnetic radiation emitted by the discharges using UHF sensors [15]. Nevertheless, the detection and the subsequent identification of the problematic points are difficult when there is more than one deterioration process, and even more so when their activity comes from similar defects. Hence, the motivation to use ICA to separate each source in the post-processing. In this paper, we propose an experiment to detect two sources of the same type of discharge and a reference to assess the suitability of ICA. Each source consists of a polyethylene sheet placed between two cylindrical electrodes. These test objects produce surface discharges in the interface between one of the electrodes and the dielectric sheet. When high voltage is applied, the component of the electric field parallel to the sheet ionizes the surrounding air and makes it conductive, triggering an electron avalanche. This energy pulse emits in a broadband of frequencies, including the UHF band.

**Figure 1:**
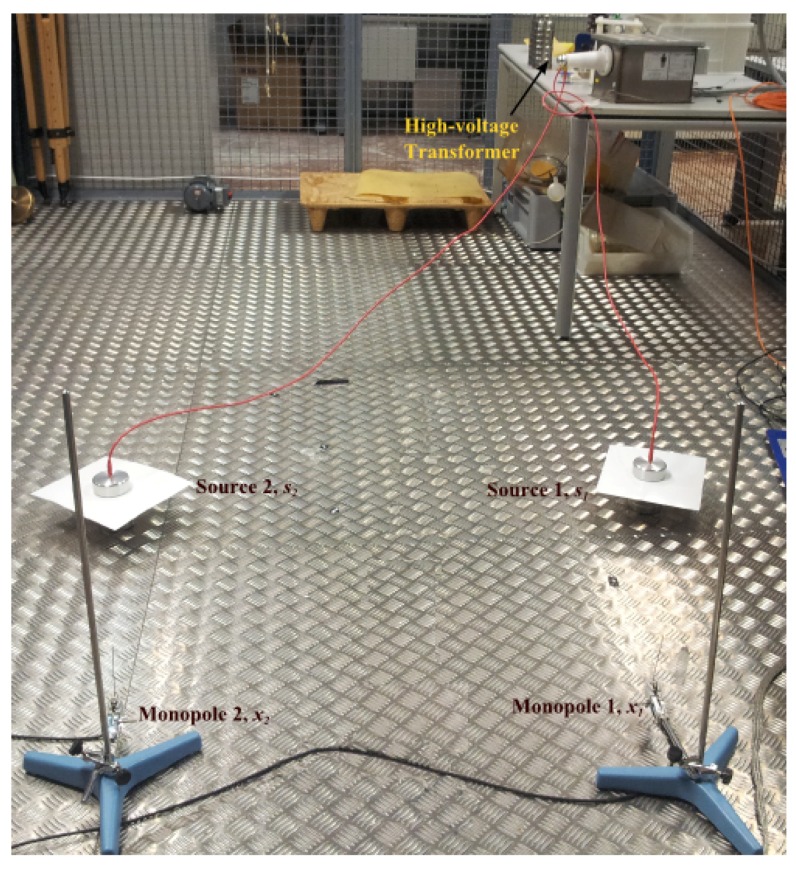
Picture of the experimental arrangement showing the two sources and the two antennas (UHF sensors) for partial discharge (PD) detection.

The test objects are excited with a high-voltage transformer at 6 kV obtaining controlled PD activity (see Figure 1). The electromagnetic radiation of PD will be detected and registered with UHF sensors located around the test objects. The sensors are two simple and inexpensive monopoles that have a resonant response at λ/4, λ being the wavelength of the main frequency. Setting the length to 10 cm (λ is then 40 cm), the resonant frequencies are 750 MHz and its multiples, and it is possible to acquire all types of partial discharges at relatively far distances without the use of a wideband amplifier. The monopoles are connected to a Tektronix DPO7254 8-bit, 40 GS/s, 4-channel oscilloscope through coaxial cables with the same length.

**Figure 2:**
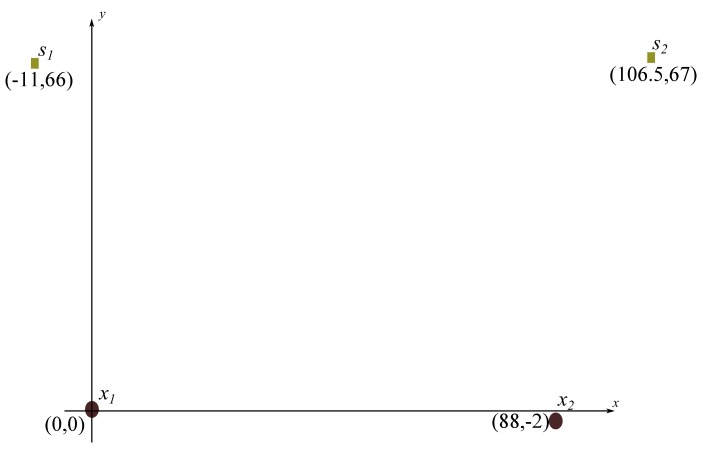
Positions of sources and sensors. Cartesian coordinates are in centimeters.

The positions of the antennas are chosen in such a way that the detected signal is in the far-field region of the PD electromagnetic activity. Figure 2 shows the positions of the antennas and sources used in the experiment, where $x_{1}$ and $x_{2}$ correspond to the monopole antennas with the positions indicated in Cartesian coordinates, and $s_{1}$ and $s_{2}$ are the test objects.

The experiments were carried out as follows. First, each source was excited individually to verify that they produced controlled and permanent activity at the same voltage level. These signals also help to train the automatic classifiers used in the validation stage (see Section 4). Then, both sources were excited simultaneously and the registered pulses were delivered to the ICA algorithm to separate them. In each experiment, each sensor registered a series of 100 consecutive pulses with a duration of 100 ns, giving a total time interval of 10 μs.

## 3. ICA for PD Activity Signal Processing

The UHF sensors, $x_{1}$ and $x_{2}$, indiscriminately capture the signals coming from the two active PD sources, $s_{1}$ and $s_{2}$, when there is a simultaneous excitation. The result is a combination of signals that does not give information suitable for monitoring the equipment effectively. To undertake this challenge, an approach based on blind source separation (BSS) is proposed using a separation method for retrieving the original source signals when there is little or no knowledge about the sources. In this work, we follow the ICA implementation of Choi et al. [16].

**Figure 3:**
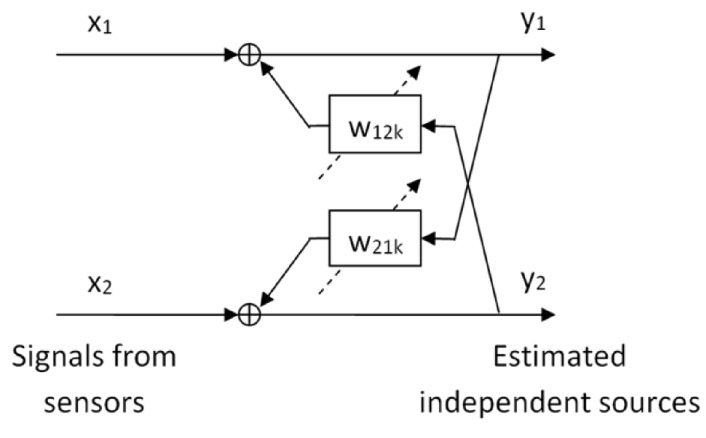
Schematic representation of the independent component analysis (ICA) algorithm. $W_{12k}$ and $W_{21k}$ are two recurrent filters whose coefficients are determined using an unsupervised learning rule.

Figure 3 shows the separation scheme of the algorithm for two PD sources. The measured signals, $x_{1}\left(t\right)$ and $x_{2}\left(t\right)$, containing a combination of the two sources, $s_{1}\left(t\right)$ and $s_{2}\left(t\right)$, are processed by the recurrent finite impulse response (FIR) filters $W_{k}$. The aim of this processing is to estimate and place a single source, $y_{i}\left(t\right)$, in each channel. The scheme presented in Figure 3 represents the architecture of the recurrent network of separation and can be expressed by a discrete time equation as follows:

(1) $$y\left(t\right)=x\left(t\right)+\sum_{k=0}^{L}W_{k}y\left(t-k\right)$$

where $y\left(t\right)={\left[y_{1}\left(t\right),y_{2}\left(t\right)\right]}^{T}$ and $x\left(t\right)={\left[x_{1}\left(t\right),x_{2}\left(t\right)\right]}^{T}$ are the estimated sources and the signals measured by the sensors, respectively; $W_{k}$ is the matrix of filter coefficients at delay *k* and *L* defines the maximum order of the FIR filters. The filters consist of 2 × 2 matrices with zeros in the diagonals:

(2) $$W_{k}=\left[\begin{array}{cc} 0 & w_{12k}\\ w_{21k} & 0 \end{array}\right].$$

These matrices model the room effects (boundaries, floor, walls, ceiling and other obstacles) on the UHF signals that arrive to the sensors. Therefore, the length of the filters must be long enough to evaluate the direct wave front from the PD source and its possible reflections. The value of *L* can be determined as follows:

(3) $$L=\frac{l\cdot f_{s}}{c}$$

with $f_{s}$ being the sampling frequency and *c* = 3 × 10${}^{8}$ m/s (the speed of light). *l* has an upper limit in the maximum path difference that a reflected wave can travel without being attenuated under the detection limit of the sensor.

The determination of the coefficients of the filters follows an iterative process based on optimizing an objective function that maximizes the independence between $y_{1}\left(t\right)$ and $y_{2}\left(t\right)$. The learning algorithm for updating the separation demixing filter coefficients $W_{k}$ for each iteration *n*, is:

(4) $$W_{k}\left(n+1\right)=W_{k}\left(n\right)-\eta \left[I-W_{0}\left(n\right)\right]\varphi \left(y\left(t\right)\right)y{\left(t-k\right)}^{T}$$

where **I** is the identity matrix and η>0 is the learning rate. $W_{0}$ is the matrix for k=0 and it is equal to $\left[\begin{array}{cc} 0 & 1\\ 1 & 0 \end{array}\right]$. $\varphi \left(y\right)={\left[\varphi_{1}\left(y_{1}\right),\varphi_{2}\left(y_{2}\right)\right]}^{T}$, is an element-wise nonlinear function selected depending on the probability density function (PDF) assumed by the unknown sources. The PDF of the signal measured by the UHF sensors has been assumed as super Gaussian, so the function proposed by Bell and Sejwnoski in [17] has been used to separate the PD sources:

(5) $$\varphi_{m}\left(y_{m}\right)=\tanh\left(y_{m}\right).$$

The source estimation procedure utilizing ICA can be summarized in the following steps:

1. Set the parameters of the algorithm: length of filter, *L*, learning rate, η, and number of iterations, *n*.
2. Apply Equation (1) in a block of size equal to the samples of each measurement.
3. Use Equation (4) to update $W_{k}$.
4. Repeat steps 2 and 3 until the number of the chosen iterations is reached.

## 4. Validation of ICA Through Automatic Classification

ICA decomposes the measured signals, $x_{j}\left(t\right)$, into independent components, $y_{i}\left(t\right)$, individually per output channel. Ideally, each output channel will align with a single source $s_{i}\left(t\right)$, meaning that ICA has actually separated the contribution of each source. Therefore, the purpose of this section is to present a methodology based on machine learning that enables evaluation of the degree of matching between the $y_{i}\left(t\right)$ and the original sources. An optimum test would require a complete statistical characterization of each source, followed by a measure of the likelihood of each output signal being generated by the corresponding source. Since this complete characterization is impractical, it turns out to be more realistic to design the test using machine learning.

The proposed test consists in training an automatic classifier with signals recorded separately at each source (without interferences from other sources) and evaluating the classifier with the signals reconstructed by ICA. A high classification accuracy will indicate that ICA is performing a correct separation in components that can be matched with the original sources.

An automatic classification method essentially constructs a function f(z) that takes as input a vector z formed with the features that define each observation and gives an output that indicates the correct class of this observation. To continue with the case presented along the previous sections, we consider two output classes, $s_{1}$ and $s_{2}$ (each PD source determines a class). The usual convention is to assign a positive label (c=+1) to the instances of one class and a negative label (c=−1) to the instances of the other class. With respect to the feature vectors z, we use the normalized power spectrum density (PSD) of each recorded pulse since the shape of the spectral envelope is informative to discriminate the source of PD [14].

From all the available automatic classification techniques we focus on kernel methods (KMs) [18] since they are considered the de facto standard within the machine learning community. KMs rely on the use of a kernel function to construct f(z). A kernel function $\kappa (z_{p},z_{q})$ can be regarded as a similarity measure between data samples $z_{p}$ and $z_{q}$ in a certain feature space: if $z_{p}$ and $z_{q}$ are very similar (different), then $\kappa (z_{p},z_{q})$ will take a high (low) value. In the most widely used kernel functions, the values of $\kappa (z_{p},z_{q})$ range between 1 ($z_{p}=z_{q}$) and 0 ($z_{p}$ and $z_{q}$ are orthogonal). In a classification setting, two samples from the same class are expected to yield a higher value of the kernel function than two samples from different classes. Therefore, most KM classifiers consist of a linear combination of kernel functions centered on samples that are relevant to the definition of the support of each class. This way, a sample $z_{p}$ of the positive class would yield a higher value in the kernels centered on positive samples, thus pushing the linear combination in $f(z_{p})$ towards a final positive value. Conversely, a sample $z_{n}$ of the negative class would yield a final negative $f(z_{n})$.

In this paper, we use a kernel function based on exponentiating a symmetrization of the discrete Kullback–Leibler (KL) divergence between $z_{p}$ and $z_{q}$ [19]:

(6) $$\kappa \left(z_{p},z_{q}\right)=\exp\left\{-0.5\cdot \left(\mathrm{KL}\left(z_{p}∥z_{q}\right)+\mathrm{KL}\left(z_{p}∥z_{q}\right)\right)/\sigma \right\},$$

with

$$\mathrm{KL}\left(z_{p}∥z_{q}\right)=\sum_{d=1}^{D}z_{p}^{d}\log\frac{z_{p}^{d}}{z_{q}^{d}},$$

providing that $z_{q}^{d}=0$ implies $z_{p}^{d}=0$. Scalars $z_{p}^{d}$ and $z_{q}^{d}$ are the d-th components of vectors $z_{p}$ and $z_{q}$, respectively.

The rationale behind the use of this kernel is the following. Each z is normalized to unit area and can therefore be regarded as a discrete probability (all their components are positive and add up to one). A natural measure of divergence among discrete probabilities is the KL divergence, that needs symmetrization to be considered a proper distance. Likewise, the exponentiation of a distance becomes a kernel. Parameter σ determines the spatial resolution of the kernel. Intuitively, the KL kernel somehow measures the overlap between the two PSDs.

We have selected two KM classification technologies that potentially present complementary views of the classification problem for the validation of ICA: the support vector machine (SVM) [13] and a naive classifier consisting of assigning each sample to the class of the closest mean. We talk about complementary views because the former stresses those features that make samples in one class look different from the samples in the other class, while the latter stresses those features that make instances within the same class look similar (and hopefully different from instances in the other class).

The SVM classification of a test sample z is based on evaluating the sign of a linear combination of kernels that can be split in two terms:

(7) $$o\left(z\right)=\sum_{p\;\mathrm{with}\;c_{p}>0}\alpha_{p}c_{p}\kappa \left(z_{p},z\right)+\sum_{n\;\mathrm{with}\;c_{n}<0}\alpha_{n}c_{n}\kappa \left(z_{n},z\right)+b.$$

The first sum is the weighted similarity of sample z with the support vectors (SVs) of the positive class. Analogously, the second sum is the weighted similarity of z with the SVs of the negative class. The SVs are critical samples that define the classification boundary. They, together with the coefficients of the linear combination $\alpha_{p}$, $\alpha_{n}$, are determined after an optimization [13].

With respect to the naive classification method, we calculate the mean of each class as:

$$m_{1}=\frac{1}{N_{p}}\sum_{p\;\mathrm{with}\;c_{p}>0}z_{p};\;m_{-1}=\frac{1}{N_{n}}\sum_{n\;\mathrm{with}\;c_{n}<0}z_{n}$$

where $N_{p}$ is the number of positive examples in the training set (those with label $c_{p}>0$) and $N_{n}$ is the number of negative examples. The classification of a test sample z is as follows:

(8) $$f\left(z\right)=\mathrm{sign}\left(\kappa \left(m_{1},z\right)-\kappa \left(m_{-1},z\right)\right).$$

## 5. Experimental Results

**Figure 4:**
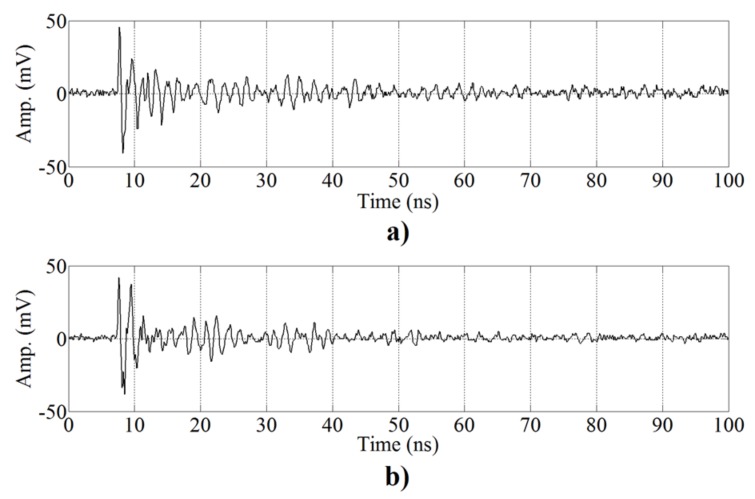
Representative UHF signals from the two surface test objects. (**a**) $s_{1}\left(t\right)$ and (**b**) $s_{2}\left(t\right)$.

The performance of the proposed separation algorithm is tested with the measurements obtained following the setup described in Section 2 with both sources excited simultaneously. The results obtained applying the ICA separation algorithm will be compared with stand-alone signals registered from each source by the nearest sensor to obtain cleaner pulses with enough amplitude. Pulses from $s_{1}$ are recorded by sensor $x_{1}$, while pulses from $s_{2}$ are recorded by $x_{2}$ (see Figure 4). It is important to stress that these stand-alone measurements of each source will be only used to train the automatic classifiers for the validation.

### 5.1. ICA Results

Two series of combinations of signals from both sources, $x_{1}\left(t\right)$ and $x_{2}\left(t\right)$, are processed by the proposed algorithm. An adequate separation of the signals into the original UHF sources is made after several simulations and is achieved by the parameters summarized in Table 1. The length of the filter, L=50, was obtained for a maximum propagation l=1.5 m and $f_{s}=10$ GS/s. The weights, $W_{k}$, for k=1,…50, are initialized to zero. Both the learning rate and the number of iterations are obtained empirically based on previous experimental measurements done in the radio-frequency localization of PD sources [12].

**Figure 5:**
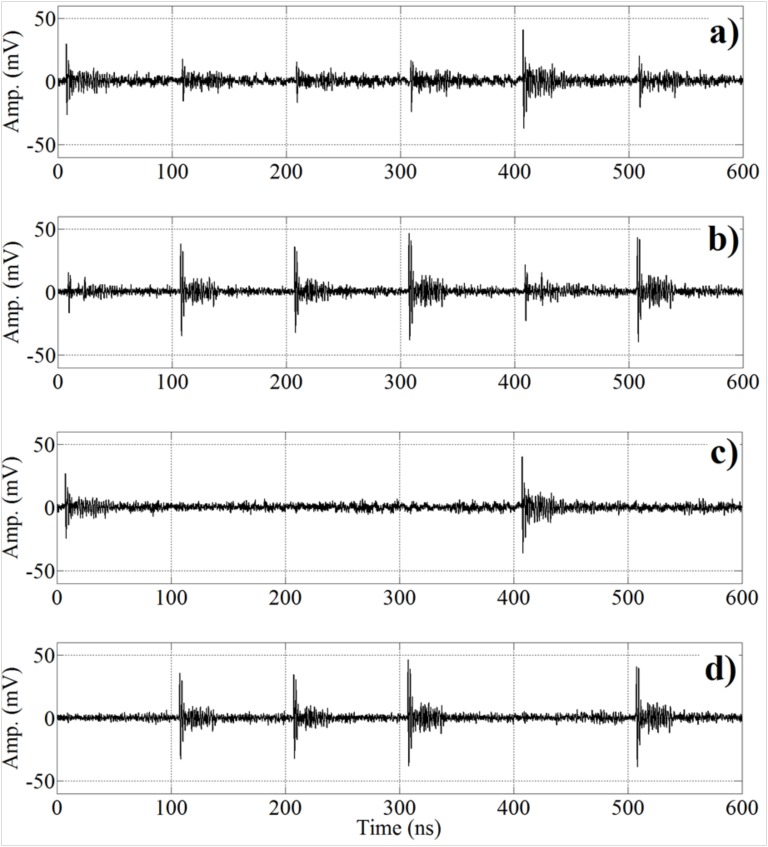
Example of the signals involved in the different stages of the algorithm. (**a**) UHF signal at sensor $x_{1}$, (**b**) UHF signal at sensor $x_{2}$, (**c**) Estimated signal $y_{1}\left(t\right)$, and (**d**) Estimated signal $y_{2}\left(t\right)$.

The outputs obtained are the source estimations, $y_{1}\left(t\right)$ and $y_{2}\left(t\right)$, that correspond to $s_{1}\left(t\right)$ and $s_{2}\left(t\right)$, respectively. Figure 5 displays examples of the different signals involved in all the stages of the proposed algorithm. Figure 5a,b presents six consecutive pulses registered by $x_{1}$ and $x_{2}$, respectively. It can be observed that there is no superposition or mixing process in the signals owing to the short duration of the UHF signals, (tens of nanoseconds). These signals are processed by the ICA algorithm and the results are shown in Figure 5c,d, corresponding to the source estimations, $y_{1}\left(t\right)$ and $y_{2}\left(t\right)$, respectively. In each estimation, there are two types of pulses, one with high energy, that corresponds to the signals of the source; and the other with low energy, that corresponds to residual signals that remain after the separation process. After the separation process, the two automatic classification methods explained above are used to test if the separation given by ICA is correct.

### 5.2. Validation with Supervised Classification

In order to corroborate the suitability of the ICA algorithm reliably and systematically, we trained supervised classifiers to discriminate the signals coming from the two PD sources and to use them to separate the outputs of the ICA. Since the kernel function is a similarity measure, it is expected that the kernels between the signals coming from ICA and the training pulses of the correct class follow a distribution close to the one followed by the training pulses of the correct class themselves.

Following the scheme of Figure 3, input signals are decomposed into two components by ICA, and therefore the output for each input signal is double. Each channel is processed individually with all the classifiers, i.e., we check the matching of each channel to each source and make a final decision based on these classifications.

As mentioned before, two different classification techniques have been used to obtain robust results. These are SVM and similarity with class mean.

With respect to the selection of the hyperparameters of the algorithms, we have followed a very standard approach. The SVM regularization parameter *C* is selected in a logarithmic scale between 10${}^{-4}$ and $10^{4}$. The width of the kernel is selected in a logarithmic scale between 0.0025 and 10. The tuning of both parameters is carried out by a ten-fold cross validation in a grid search. Note that the test pulses are never involved in the tuning of these parameters, since they were acquired separately from the training pulses.

The first outcome of the validation is that ICA is able to separate each input into two separate components $y_{1}$ and $y_{2}$, and each component is always assigned to the right source, i.e., pulse $y_{1}$ is classified as $s_{1}$ and pulse $y_{2}$ is classified as coming from $s_{2}$ in all the test cases. Since the probability of both sources being simultaneously active during the same pulse is negligible due to the nature of partial discharges, it turns out that, independently of the amplitude of the pulses recorded at the sensors $x_{1}$ and $x_{2}$, ICA redistributes the total energy at its input in a non-uniform split, giving more amplitude to $y_{1}$ over $y_{2}$ if $s_{1}$ is active (and to $y_{2}$ over $y_{1}$ when the active source is $s_{2}$). This can be further exploited in the validation stage to enhance the visualization of the results. Notice that since the classifiers take as input the normalized PSD of the pulses, this imbalance in the energies of $y_{1}$ and $y_{2}$ is not used in the classification (i.e., the classifier would also work if both sources are active at the same time). Therefore we can display six sets of samples in the plots:

- Pulses recorded individually from source $s_{1}\left(t\right)$ (labeled S1 in the plots).
- Pulses recorded individually from source $s_{2}\left(t\right)$ (labeled S2 in the plots). Note these two sets are exclusively used as training sets for the machine learning algorithms used in the validation.
- Pulses from the first channel of the output of ICA when the first channel has more energy than the second (labeled Y1 when Y1 > Y2 in the plots).
- Pulses from the first channel of the output of ICA, when the second channel receives more energy (labeled Y1 when Y2 > Y1 in the plots).
- Pulses from the second channel of the output of ICA when the second channel has more energy than the first (labeled Y2 when Y2 > Y1 in the plots). Notice that each member of this set has a corresponding member in the set Y1 when Y2 > Y1 (both pulses are produced simultaneously).
- Pulses from the second channel of the output of ICA, when the first channel receives more energy (labeled Y2 when Y1 > Y2 in the plots). Notice that each member of this set has a corresponding member in the set Y1 when Y1 > Y2.

**Figure 6:**
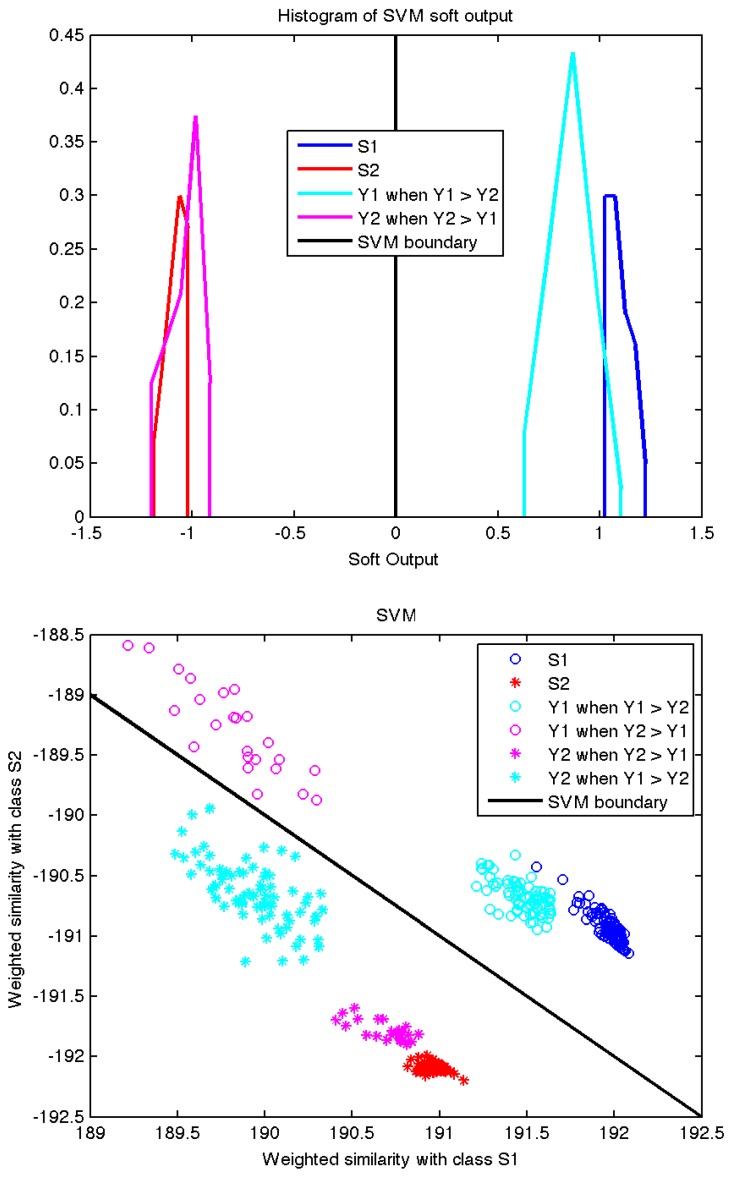
Results of the classification with support vector machine (SVM) and the Kullback–Leibler (KL) kernel. The top plot is a histogram of the classifier output for all the sets of samples. The bottom plot shows a scatter plot of training and test pulses in the 2D space defined by the two sums in Equation (7).

Figure 6 shows how training and test samples are processed by the SVM classifier. The top plot shows that the output of the classifier for those ICA outputs with more energy in the first channel is close to the output produced by the training samples recorded from PD $s_{1}$. The same applies for the ICA outputs with more energy in the second channel and the training samples recorded from PD $s_{2}$. Moreover, the 2D scatter plot shows that the shape of the pulses in the output channel with most energy lies close to the cluster formed by the corresponding training samples, clearly separated from the cluster formed by the samples of the other source. Furthermore, the shape of the pulses in the channel with less energy, residual signals, are far away from the training samples, indicating that these low energy pulses have a spectral envelope significantly different from the envelope of the training signals and the signals recovered by ICA.

**Figure 7:**
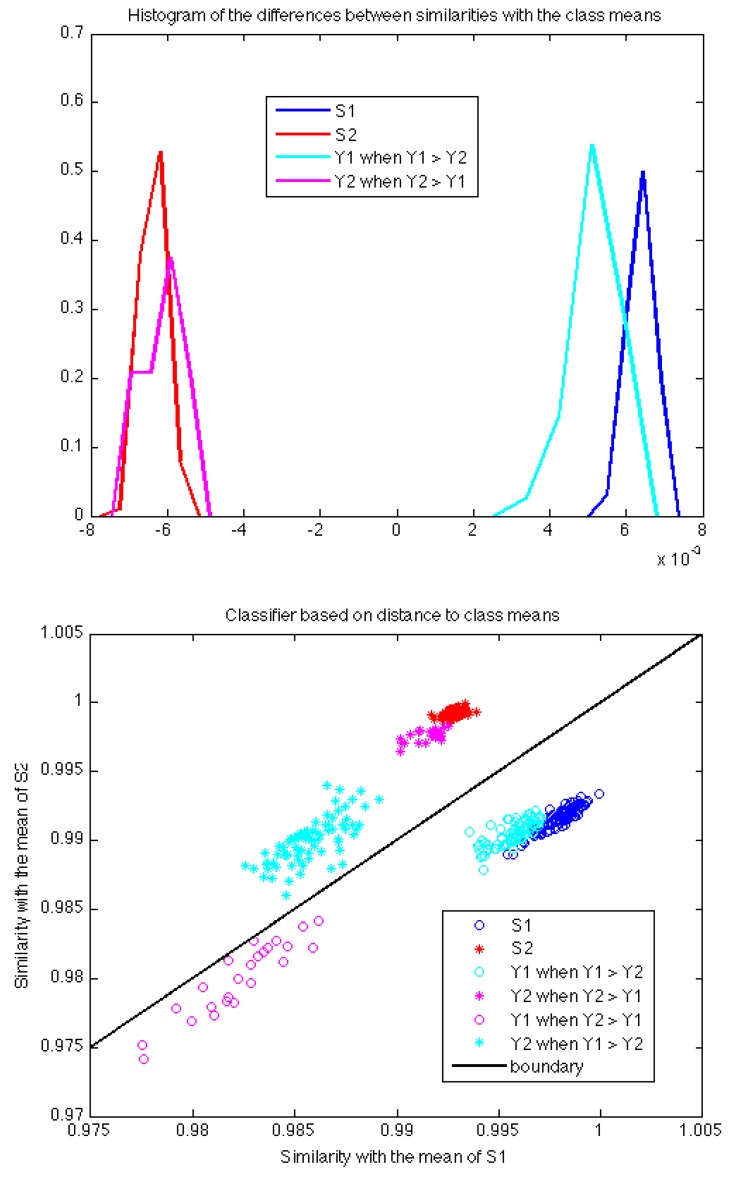
Results of the classification with the difference between the similarities of each sample and the mean of each class. These similarities are evaluated using the KL kernel. The top plot is a histogram of the classifier output for all the sets of samples. The bottom plot shows a scatter plot of training and test pulses in the 2D space defined by similarity to each class mean.

Figure 7 shows the analysis using as a classifier the difference between the similarities with the mean PSDs of each training class. This analysis completes the one performed by SVM in the following sense. The classification with the mean PSDs of each class focuses on the features that stress the similarities between instances in the same class (two instances appear to be of the same class when these features take similar values). However, the SVM classification focuses on the features that stress differences among samples of different classes (two instances from different classes would present different values with respect to these discriminating features).

Finally, the scatter plots in Figure 6 and Figure 7 confirm the intuition that the signals recovered by ICA follow the same patterns as the training signals. Although the pulses labeled as Y1 when Y2 > Y1 are classified as belonging to the same class as training pulses S1, one can check how they are in fact residual signals not generated by source S1, because they lie far away from the points labeled as S1 in the plots (the distances in the plots are given by the kernel functions). Notice how the correctly classified pulses labeled Y1 when Y1 > Y2 do actually lie very close to the training labels in both scatter plots. The same applies to source S2 and pulses labeled Y2. Therefore, we can conclude that the signals recovered by ICA are sufficiently close in spectral envelope to those training signals directly recorded at the PD source and such validation is enough to accept the future results given by the proposed algorithm in other PD measurements.

## 6. Conclusions

This paper demonstrates that the separation of PD pulses can be effectively done using independent component analysis. The results are more significant considering that the proposed method is out of the common scope of ICA which is usually applied to mixtures of signals in the time domain. Notice that the stochastic nature of PD and the short duration of the UHF pulses makes it very uncommon to have two incoming signals simultaneously in the same time window. This highlights the suitability of the ICA algorithm in other applications.

A straightforward approach to validate the results given by the ICA algorithm might have been the direct comparison of the spectra of the signals captured and labeled individually, however this would not have been applicable in this case since both signals are surface discharges and their spectra are very similar. Another approach might have been the use of time differences of arrival of the pulses to the sensors but the number of antennas should be increased and the method will fail if the sources are close to each other. Therefore, the source separation proposed by ICA has been validated with two complementary supervised classification methods, SVM and similarity with class mean. Both methods point out that each ICA channel matches with one of the sources, not only attending to the classification scores (residual signals produced in the channel matched to the non-active source could also be classified as instances of the non-active source), but also to the discriminative pattern formed by the similarities between the PSD of the signals from the ICA channels and the training signals recorded at each original source.

The supervised classification techniques are used in this paper to validate ICA when separating PD signals coming from different sources. A supervised training (i.e., SVM classifiers) will not be needed in field measurements since ICA learns how to discriminate the signals from different PD sources in an unsupervised manner (without signal examples) even when they come from different sources with the same type of insulation defect. Further research will focus on expanding ICA techniques in practical settings consisting of a set of antennas deployed in substations.

## Figures and Tables

**Table 1:** Parameters of the proposed ICA algorithm.

Names	Values
Length of filter	L=50
Learning rate	$\eta =10^{-4}$
Number of iterations	50
